# Autism spectrum disorder, functional MRI and MR spectroscopy: possibilities and challenges

**DOI:** 10.3402/mehd.v23i0.18960

**Published:** 2012-08-24

**Authors:** Kenneth Hugdahl, Mona K. Beyer, Maiken Brix, Lars Ersland

**Affiliations:** 1 Department of Biological and Medical Psychology, University of Bergen, Bergen, Norway; 2 Division of Psychiatry, Haukeland University Hospital, Bergen, Norway; 3 Department of Radiology, Haukeland University Hospital, Bergen, Norway; 4 Department of Radiology, Oslo University Hospital, Oslo, Norway; 5 Department of Clinical Engineering, Haukeland University Hospital, Bergen, Norway; 6 Department of Surgical Sciences, University of Bergen, Bergen, Norway

**Keywords:** autism spectrum disorders (ASD), fMRI, oddball paradigm, brain activation, MRS, glutamate, GABA

## Abstract

**Background:**

In this article we provide an overview of the use of the functional magnetic resonance imaging (fMRI) and MR spectroscopy (MRS) in studies of autism spectrum disorders (ASD). We moreover provide preliminary data using these measures in cases of children with ASD and healthy controls. A hypothesis was that ASD children would show aberrant brain activation in the prefrontal and parietal cortex in an oddball stimulus situation, with predictable and unpredictable deviant tone stimuli, as an index of resistance to change in the ASD children. We also hypothesized that glutamate and GABA metabolite levels would differ between the two groups.

**Methods:**

fMRI images were acquired from a GE Signa HDx 3T MR scanner, as were the MRS data. Behavioral data were acquired as response accuracy to the deviant tone stimulus. The tone stimuli were presented in a standard fMRI ON-OFF box-car paradigim.

**Results:**

The fMRI results showed reduced brain activation in the ASD cases compared to the controls, preferably in the inferior and superior frontal gyrus, posterior temporal lobe, and superior and inferior parietal lobule. These areas make up an effort mode network (EMN), being activated in response to cognitive effort. The MRS results also showed differences between the groups.

**Discussion:**

The results are discussed in a theoretical framework of resistance to unexpected changes in the environment in ASD children, and how this could have a neurobiological underpinning. The results are also discussed in relation to the brain-gut link, and the possibility that ASD may have a microbial link. A limitation with the study is the few cases reported and the preliminary quality of the results.

## Introduction

The present article is an extended abstract of a talk given at the *Nobel Forum Autsim Day: The gut and the brain, with focus on autism spectrum disorders* (*ASD*) at Karolinska Institutet, Stockholm, Sweden on 7 May 2012. The talk was an invited presentation regarding the use of advanced magnetic resonance imaging (MRI) to show neuronal correlates of ASD. This article thus gives an introductory overview of the basics of functional MRI (fMRI), based on the blood-oxygenation level dependent (BOLD) contrast phenomenon (1), and magnetic resonance spectroscopy (MRS), which allows for quantification of regional concentrations of brain metabolites, acting as synaptic transmitters. The article will provide examples of the use of these methods for the understanding of brain function and pathology, with reference to psychiatric disorders, including ASD.

## The Bergen fMRI Group

The research that is presented is being conducted by the ‘*Bergen fMRI Group*’ (see http://fmri.uib.no/ and http://www.youtube.com/watch?v=6UhfAX3RusE). The Bergen fMRI Group is a multidisciplinary research group at the University of Bergen and Haukeland University Hospital, Bergen, Norway, that pioneered the use of functional MRI (fMRI) imaging in the Nordic countries in the mid-1990s. The group has over the years published numerous articles on fMRI, including aspects of methodology and measurements (e.g. 2–4), issues in cognitive neuroscience (e.g. 5–10) to the use of fMRI in clinical assessments (e.g. 11–13). Similarly, the Bergen fMRI Group has been engaged in the use of MRS to investigate the transmitter systems underlying the fMRI response (3, 43) and to evaluate the effects of pharmacological manipulations of the brain (14).

## Functional MRI

**Fig. 1:**
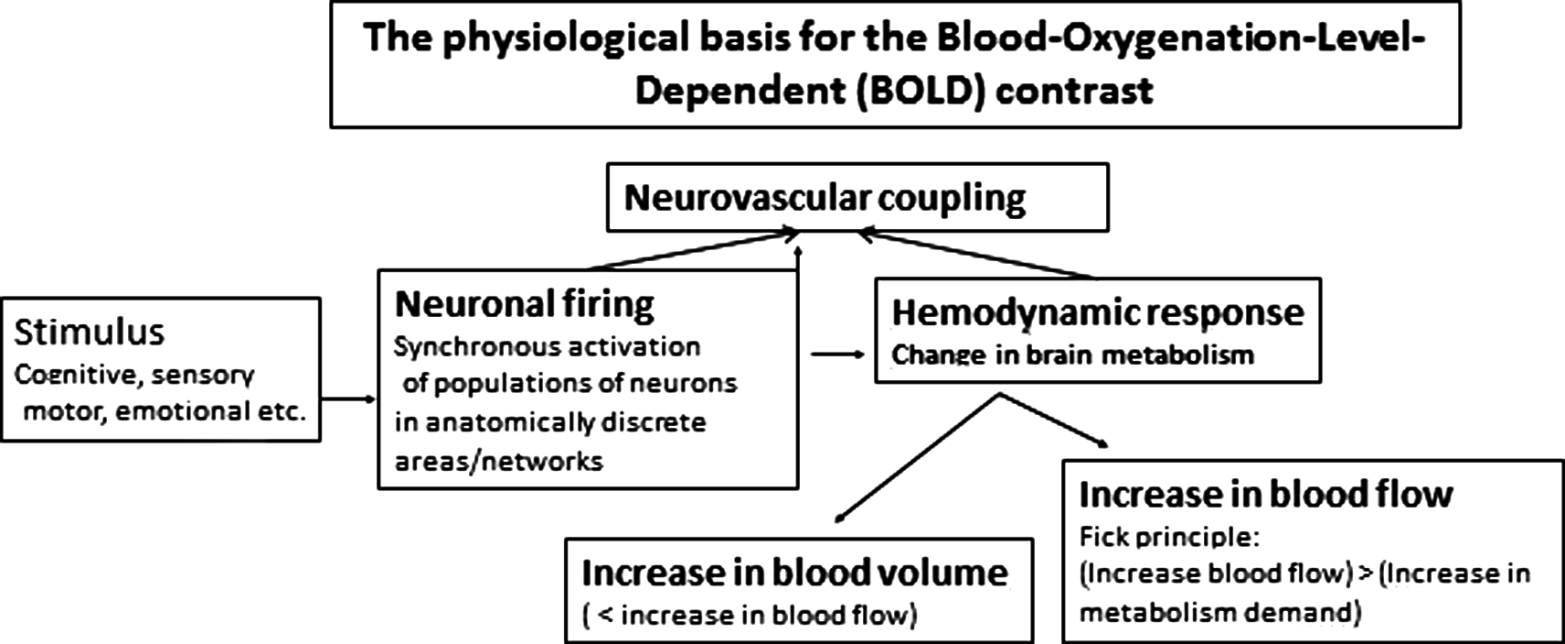
Schematic outline of the physiological principles behind the fMRI BOLD response. See text for further explanations.

fMRI is a non-invasive method used to indirectly measure neuronal activation to cognitive, motor, sensory, or emotional tasks, thus allowing for regional localizations of a specific function (15). fMRI is based on the BOLD contrast, which reflects the relationship between change in neuronal activation and the corresponding change in oxygen extraction and blood flow. Oxygen is a key component in neuronal metabolism and is transported to the brain and the neurons with the brain blood supply, as oxyhemoglobin, bound to hemoglobin. Performing a cognitive task, e.g. a memory or attention task, will result in a change in neuronal activity and corresponding metabolism that is anatomically constrained to brain areas involved in the specific task. Since oxygenated blood has diamagnetic properties, while deoxygenated blood has paramagnetic properties, with increased MR signal susceptibility, then areas rich in oxygen will result in a stronger MR signal than areas deprived of oxygen. Figure 1 shows the principles behind the BOLD activation response.

Thus, fMRI provides a measure of changes in blood flow and oxygen extraction, with anatomical specificity, which will reflect increases in metabolic demands that correlates with increases in neuronal firing and synaptic activity (16). By comparing BOLD images acquired during the performance of a cognitive task (ON block) with BOLD images acquired during a resting period without the cognitive task (OFF block) and subtracting the signal intensities during task-absence from the images acquired during task-presence, on a voxel-by-voxel basis throughout the entire brain volume, it is possible to identify brain areas (voxels) uniquely activated to a specific cognitive task.

A full brain scan is typically acquired every second or third second and averaged across time, subjects, and ON/OFF conditions, which will result in several 100 GB of data for a single subject. The subtracted averaged BOLD signal is finally subjected to tests for statistical significance on a voxel-by-voxel basis, typically with *t* tests. Since the brain volume will contain about 30–50,000 voxels (depending on the sequence parameters), with a voxel size of 3.5×3.5×5 mm^3^, a corresponding number of *t* tests will be calculated, resulting in a mass-significance problem. This is typically handled by adding statistical procedures that correct the significance level for the number of *t* tests being performed. fMRI is a key method to create a kind of ‘mental maps’ in the brain by showing how different cognitive functions and processes are localized to specific regions and areas in the brain, sometimes also being connected into functional cortical networks, also called psychophysiological interactions (cf. 17).

## Limitations and challenges of fMRI

Although fMRI can be said to have revolutionized the neurosciences, and being behind the development of cognitive neuroscience as a new branch of the neuroscience family, there are also several limitations and pitfalls associated with an fMRI investigation. First of all, fMRI does not measure neuronal activity in itself but is an indirect measure of such activity. Second, it is important to supplement fMRI data with performance data, e.g. how accurate or fast a subject performs on a task. This is especially important when comparing clinical groups, as, for example, a group of ASD children and a healthy control group. Third, fMRI data are typically analyzed as group averages; thus, it is currently not possible to use fMRI to diagnose individual patients (more recent statistical advances have however provided a possible opening toward such use). The distinction between a focus on groups versus individuals is similar to the distinction between statistical and clinical significance (cf. 18) where statistically significant differences between a clinical and control group not necessarily also means that the difference is clinically significant. Fourth, fMRI data are very sensitive to head movements that distort the unique anatomical localizations. Moderate head movements can be handled with data preprocessing algorithms, but with large rotations and translation of the head while in the scanner, the data will be compromised. Fifth, but not the least, fMRI data cannot in itself prove causality, and such data can only state if a certain brain region is involved in a specific cognitive process, but not whether it is necessary and/or sufficient for the process in question. For such conclusions, lesion data and animal models are still the ‘gold standards’.

## An effort mode network

**Fig. 2:**
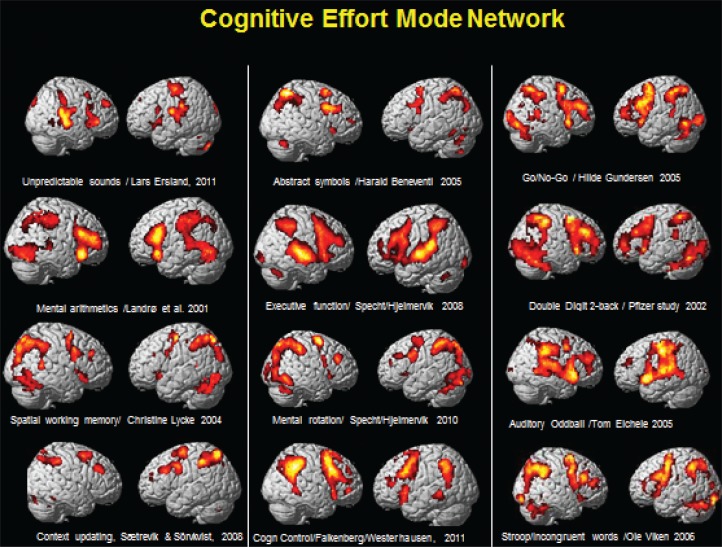
Example of neuronal activations as measured from fMRI BOLD responses (red/yellow areas) and visualized on a 3D structural MR image. The figure shows commonalities in network activation across studies and experimental manipulations, pointing toward a common effort mode network, which is dependent on an above threshold cognitive load in need of cognitive effort to be processed. See text for further explanations. The figure has been composed by Kenneth Hugdahl from several different studies conducted by the Bergen fMRI Group, with compliments to the PIs of the studies.

Figure 2 shows activations from 12 different studies conducted at the University of Bergen, Norway, over an extended time period. Although the studies used different cognitive tasks, the overall pattern of activation shows similarities with an extended frontoparietotemporal network being activated across tasks. This can be labeled an ‘effort mode network’ (EMN), which is activated whenever the cognitive challenge is above an effort threshold and which is in contrast to a resting state, or default mode network (DMN). The DMN was discovered by Raichle et al. (19) and has a more posterior localization than the EMN and is activated in the absence of an external or internal stimulus. The DMN is supposed to be downregulated whenever a cognitive task is present (either externally or internally generated). If aberrant neuronal activation is present, as in certain clinical conditions, e.g. ASD, cognitive functioning could be compromised due to neuronal interference caused by failure to downregulate the DMN and simultaneously upregulate the EMN in cognitive situations requiring effory (12).

## MR spectroscopy

**Fig. 3:**
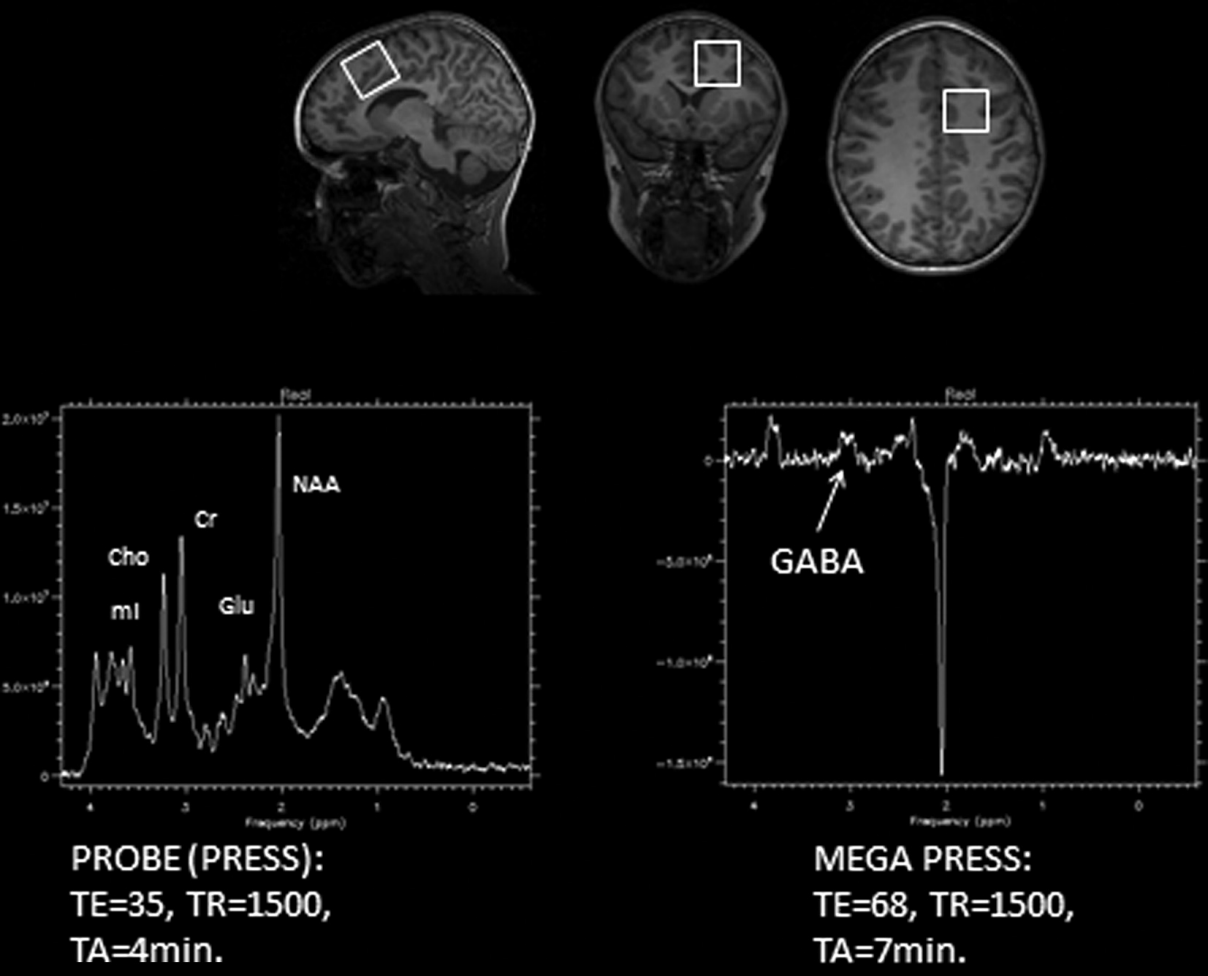
Left hand panel: example of a MR spectroscopy spectrum using a PRESS sequence showing some of the observed brain metabolites, such as *N*-acetyl aspartate, glutamate (Glu), creatine, choline), and myoinositol. Right hand panel: MR spectroscopy showing the metabolite GABA when using a MEGA PRESS sequence with and without GABA editing pulse. The upper row shows localization of the 3×3×3 cm voxel (white squares) in the anterior cingulate cortex in the frontal lobe for the MRS measurements. Values for TE and TR are in ms.

While the MRI techniques described above provide information about brain function and anatomical structure, MRS provides information about brain tissue chemical integrity. The most commonly used MRS technique probes the signal and chemical shift from the hydrogen molecule, H^1^. H^1^-MRS can be used for quantitative measurements of relative or absolute concentrations of biologically important chemical compounds such as *N*-acetyl aspartate (NAA), creatine (Cr), choline (Cho), myoinositol (ml), glutamate (Glu), and glutamine (Gln), to give some examples. The signal from these metabolites are localized spectrally between the water and lipid peaks but are much weaker than the signal from water and lipids that gives the structural MR images. In order to get good enough signal-to-noise ratio, the voxel size has to be increased to >1.0 cm^3^. A point resolved spectroscopy sequence (PRESS) (20, 21) technique using the MR gradient coils to acquire localized signals from a single voxel (SV) in the brain is used. For detecting low concentration coupled spin systems such as gamma-aminobutyric acid (GABA), a spectral editing technique called MEGA PRESS (22, 23) is used. Because of the low concentration, it is necessary to measure from a larger voxel to detect GABA reliably. In the study presented here, a SV PRESS and a MEGA PRESS sequence were used from the same voxel with dimension 3×3×3 cm (see Fig. 3). The PRESS sequence have TR = 1500 ms, TE = 35 ms, and an acquisition time of 4 min. The MEGA PRESS have TE = 68 ms, TR = 1500 ms, and an acquisition time of 7 min.

A spectrum plot that shows the peak values of the typical metabolites, including Glu, observed with the PRESS sequence are displayed in Fig. 3, left hand panel. The right hand panel shows the GABA peak value observed with a MEGA PRESS sequence with two successive scans, with and without a GABA editing pulse. Subtraction of the two scans will result in selective detection of GABA sensitive resonance, while at the same time removing other resonance peaks not affected by the editing pulse.

Relatively few studies exist with MRS measurements compared to the great amount of structural MRI and fMRI studies on ASD (cf. 24 and 25). However, Ipser et al. (26) reported in a meta-analysis based on 20 studies with N = 852 of increased Cr levels for ASD children compared to controls in global gray matter, with no differences between the groups in frontal lobe areas. They also reported decreased NAA levels in frontal cortex and in the anterior cingulate cortex (ACC) in younger children in general, as a marker of brain development. Another review (27) focused on the role played by the excitatory and inhibitory metabolites, Glu and GABA, respectively, and the authors suggested that studies of neurochemistry balances in the ASD brain should be integrated with genetic, behavioral, and neuroimaging studies in order to move the knowledge of factors underlying ASD further ahead. A few other studies have found distinct patterns of chemical alterations in gray matter in an ASD group compared to normally developing children and children with delayed development but with no symptoms of autism (28, 29). These findings provide evidence of abnormalities in cortical gray matter early in life in autism that are distinct from abnormalities in children with non-autism development delay. Furthermore, these findings suggest that the primary triggering event of autism might be neuronal in the cerebral cortex and that the white matter changes described earlier are secondary to that event. Relative few MRS studies have furthermore looked at changes in cerebellum that will affect neuronal pathways. Our own BOLD fMRI results have revealed changes in activation in areas in the cerebellum; thus, measures of metabolites in the cerebellum may be a potentially important new avenue in ASD research.

## The Bergen autism spectrum disorder study

The Bergen fMRI Group initiated a fMRI and MRS study on ASD in 2011 with Lars Ersland, PhD, as principal investigator, and with Mona Beyer, MD, PhD, Maj-Britt Posserud, MD, PhD, Åsa Hammar, PhD, and Kenneth Hugdahl, PhD as collaborators, and with Maiken Brix, MD, as PhD student. The fMRI and MRS study has partial funding from the Helse-Vest Funding Agency in Bergen and is approved by the Regional Committee for Medical Research Ethics in Western Norway (REK Vest).

ASD includes a range of complex pervasive neurodevelopmental disorders defined on clinical basis by impaired social interaction, by communication difficulties (both verbal and non-verbal), and by repetitive, restricted, or stereotypic behavior. In the DSM-IV (30) classification, autism is further assigned to five subtypes based on number and particular kinds of symptoms, severity, age of onset, levels of functioning, and challenges with social interactions. Autism, also called classical ASD, is the most severe form of ASD, while other conditions along the spectrum include a milder form known as Asperger's syndrome, the rare condition of Rett syndrome, childhood disintegrated disorder and pervasive developmental disorder not otherwise specified. There are three to four times more boys than girls diagnosed with autism. There are no definitive biological markers of autism, and the diagnosis relies on the recognition of a range of behavioral symptoms that vary from case to case. Some people with ASD have a vast vocabulary and grammar, others use only standardized, repetitive sentences, and still others do not talk at all. People with ASD do not only show difficulties in expressing language but also understanding communication of others often goes literally and context independent.

## Cognitive deficits

From a cognitive point of view, ASD has two core characteristics: inability to engage in social interactions and an inability to adequately read the mind and intentions of others. The first deficit is traditionally operationally defined as a deficit in face processing, i.e. the reading of the intentions or emotions facially expressed by others as shown in facial expressions (31, see also 32 for studies on response to pictures of emotional facial expressions in healthy individuals). The second deficit is traditionally operationally defined as a deficit in ‘theory-of-mind’ tasks (33). Deficit in face processing has been shown to relate to discriminate between pictures of familiar and unfamiliar faces (e.g. 34) and to discriminate between emotional facial expressions (35). Most of these studies are behavioral studies, that is, they have used behavioral measures that would not reveal if the deficits in social interaction and face processing, and in the ability to mentally ‘put oneself in the clothes of another person’ have any biological correspondences. fMRI, as described above, could, however, provide a window into the brain of ASD children and fMRI images could be acquired while the child is performing these tasks while in the scanner and the data then analyzed as contrasting signal intensities in the task-presence and task-absences conditions.

Several fMRI studies have revealed that ASD children show different patterns of activation compared to control children, notably reduced activation in the prefrontal and parietal cortices that are critical areas for higher cognitive functions, like theory-of-mind (see 24 for a meta-analysis). The dorsal portion of the ACC seems to play an important role in social cognition (24), with aberrant activations seen in children with ASD. Interestingly, ASD children also show reduced activation in a small area in the fusiform gyrus in the posterior part of the brain (36), labeled the ‘fusiform face area’ by Kanwisher et al. (37). Thus it seems clear that ASD children show aberrant neuronal activations to cognitive and emotional tasks that engage large portions of cognitive effort or executive networks that also are engaged in social interactions.

## The experimental design

As mentioned above, many of the symptoms and behaviors seen in ASD children have been the target of fMRI studies that have shown aberrant activations compared to healthy controls. These include studies of face perception and face processing (36) and studies of social interaction and communication (see 24). A class of symptoms that have not been the target for fMRI studies is the extreme resistance to change displayed by children with autism. ASD children show signs of distress whenever unexpected events occur in their near environment (see however 44 for a very recent study addressing this question). We therefore decided to set up an experimental situation that would fit the requirements for an fMRI and MRS study and that would mimic the everyday situation of changing environments. The study is still ongoing and in a data acquisition phase; thus, the results presented in this article are preliminary and should be considered as pilot-data only.

## Resistance to change: an oddball paradigm

In experimental terms, unexpected events in a stream of expected events is typically operationally defined as an auditory ‘oddball paradigm’ where, e.g. a stream of tone pips is unexpectedly interrupted by the presentation of another tone differing in pitch, at irregular intervals. Variants of the oddball paradigm has a long tradition in electrical neurophysiology, using electroencephalography (EEG) and so called event-related potentials (ERPs) (see e.g. 38–40) where the ERP response to unexpected deviant tones produce a profound negativity in the EEG pattern. Since the oddball paradigm can be considered an experimental analog to the everyday situation of ‘when surprising things happen’, we constructed a stimulus paradigm consisting of repetitive presentations of similar tones, sometimes interrupted by a target tone with higher pitch.

**Fig. 4:**
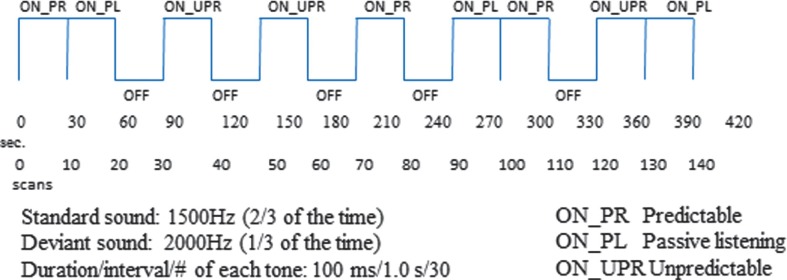
Schematic outline of the experimental design for the Bergen autism symptom disorder (ASD) fMRI study. The outline shows the alternations between ON- and OFF-blocks for the predictable (PR), unpredictable (UPR), and passive listening (PL) experimental conditions. Total scanning time = 7 min.

The instruction to the subject was to press a button held in their hand while in the MR scanner whenever they detected the deviant, target tone. The presentation of the standard and target tones occurred in three different conditions that are randomized across presentation blocks. In one condition, the target tone appears with regular intervals, producing a rhythmical pattern, called the ‘predictable’ condition. In another condition, the same two tones are presented, with identical instruction to press the response button whenever the target tone appears; but now, the target tone appears at irregular and unexpected intervals, called the ‘unpredictable’ condition. In a third condition, the subjects are instructed as for the two other conditions, but no target stimuli are presented. The hypotheses are that ASD children will show aberrant prefrontal and parietal cortex activations to the deviant sounds in the unpredictable condition and that this would go together with prolonged reaction times and reduced hit rates, also with increased omissions. The presentations of the two tones and the three different conditions follows a classic fMRI ‘box-car’ design with alternation of 30 s ON- versus OFF-blocks as a regressor variable for the statistical analysis of the BOLD data. ON-blocks are periods with stimulus presentations, and OFF-blocks are periods with no stimulus presentation. BOLD fMRI data are then acquired as the difference in signal intensity in each voxel in the brain volume for the ON minus OFF blocks, followed by statistical significance testing. Figure 4 shows the outline of the experimental design. The subjects in the Bergen ASD study are so far boys with autism and Asperger's syndrome diagnosis, between 6 and 13 years, with a similar group of healthy children as controls.

## Preliminary results

As stated above, the project is still in its infancy with ongoing data acquisition. Because we believe that combining fMRI and MRS data acquired in a single session, and with opportunities to simultaneously evaluate the effects of both excitatory (Glu) and inhibitory (GABA) neurotransmitters on the fMRI BOLD response and to compare this between ASD and healthy control children is a new and unproven pathway for unraveling the underlying neuronal mechanisms in ASD, we have chosen to present the available data here and now, despite their preliminary nature. Assessing the effects of neurotransmitters on neuronal activation may also provide an important aspect of linking microbes and gut inflammatory responses to brain function and the clinical manifestations of ASD, which was the topic of the Nobel Forum conference on ‘*the Brain and the Gut*’ at the Karolinska Institutet in Stockholm in May 2012 where the same findings were presented.

## fMRI BOLD results

**Fig. 5:**
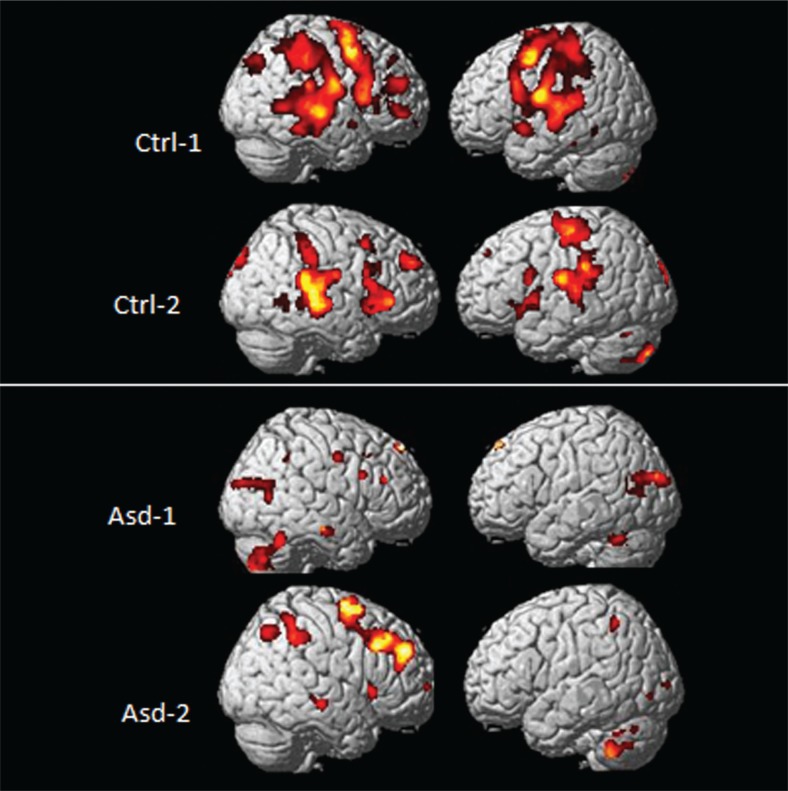
fMRI BOLD activations from two healthy controls (upper panel) and two ASD children (lower panel) as a consequence of being exposed to an unpredictable tone sequence. Significance threshold: *p*<0.001 (uncorrected), 10 voxels/cluster.

Figure 5 shows fMRI BOLD activations from two control (upper panel) and two ASD (lower panel) subjects to the target tone in the unpredictable condition that are visualized on a 3D anatomy template of the brain.

As can be seen in Fig. 5, there were significant bilateral activations in the EMN network including areas in the inferior and superior frontal gyrus, posterior temporal lobe, and superior and inferior parietal lobule in the healthy controls. The activations for the ASD children were overall reduced in comparison, particularly in frontal and temporal areas, but with greater activation seen in the cerebellum in the ASD children. The differences in activation patterns between the groups suggests reduced capacity to process cognitively demanding stimuli as when detecting an unpredictable deviant target tone in a sequence of predictable tones. This would particularly affect not only frontal and parietal areas but also temporal lobe areas, considering that the stimuli are auditory in nature. The increased activation in the cerebellum in the ASD children in comparison with the healthy control children could be a compensatory response to integrate the timing of the stimulus sequences since the cerebellum has been shown to be involved in integration and timing of sequential events (41).

## Performance results

**Fig. 6:**
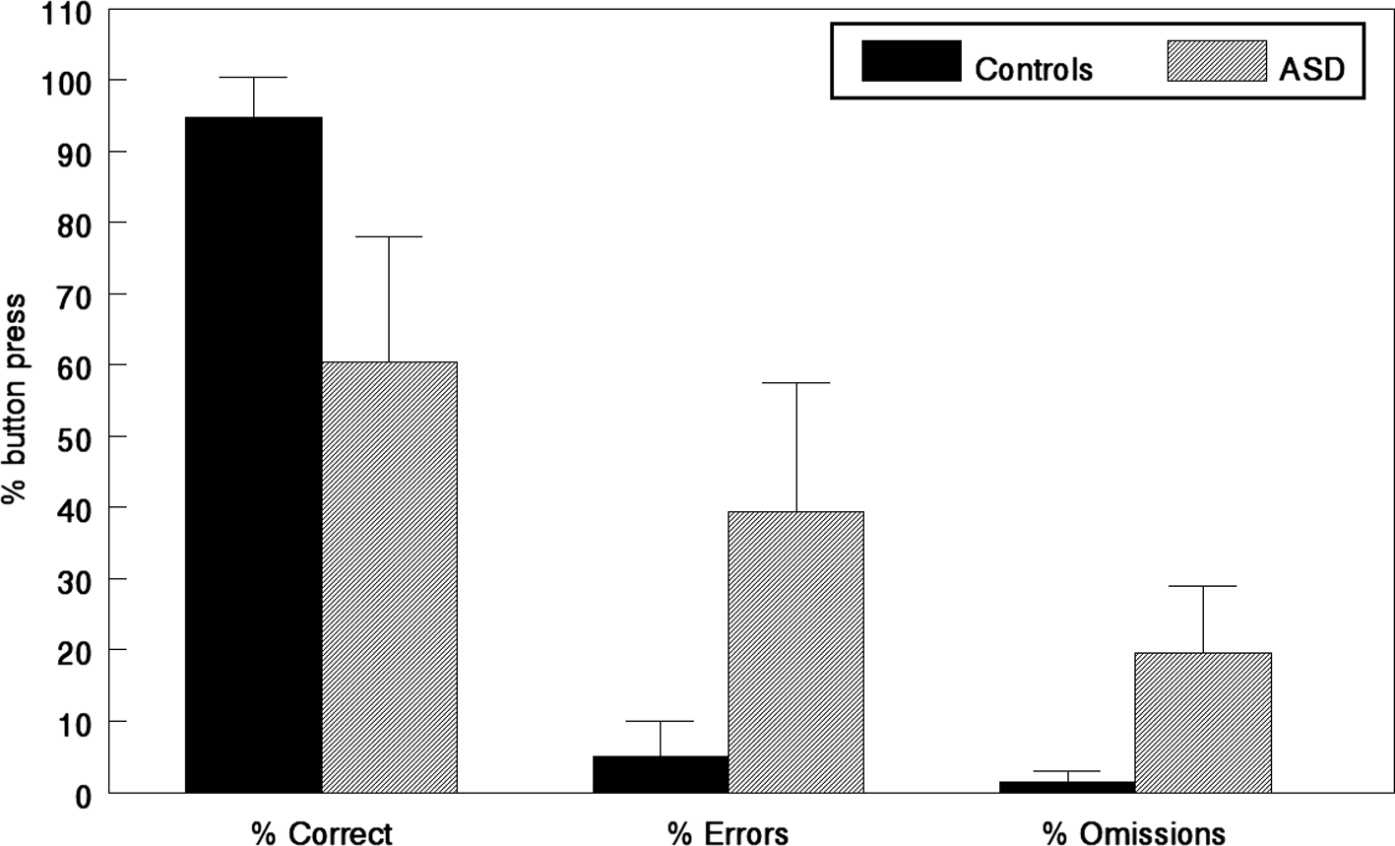
Mean percentage response accuracy for correct target stimulus responses, error responses, and omission responses for the healthy controls (solid black bars) and ASD children (grey bars) averaged across all three experimental conditions. Small vertical bars = standard error.

The reduction in activation in the EMN network in the ASD children is supported by the performance data in Fig. 6, where response accuracy was lower in the ASD children.

It is important, however, to note that the ∼60% responses by the ASD children rule out the possibility that they did not try to solve the task, and as such, the performance data validate the fMRI BOLD data.

## MRS results

**Fig. 7:**
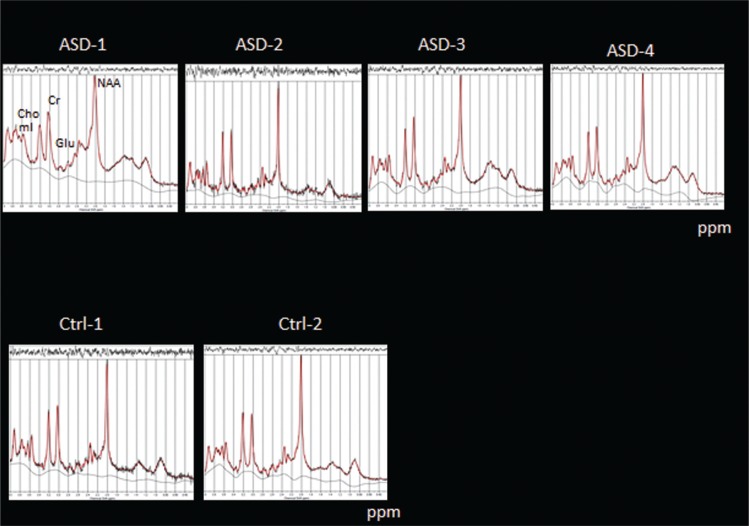
MRS measures of metabolites in four ASD children (Asd 1–4, upper panel) and two control children (Ctrl 1–2, lower panel) using a PRESS sequence targeting Glu as an excitatory neurotransmitter; ppm = parts per million.

The MRS data in Fig. 7 show a small tendency to reduced Glu amplitudes in the ASD children, but it is unsure whether this will withstand further data acquisitions and analyses.

**Fig. 8:**
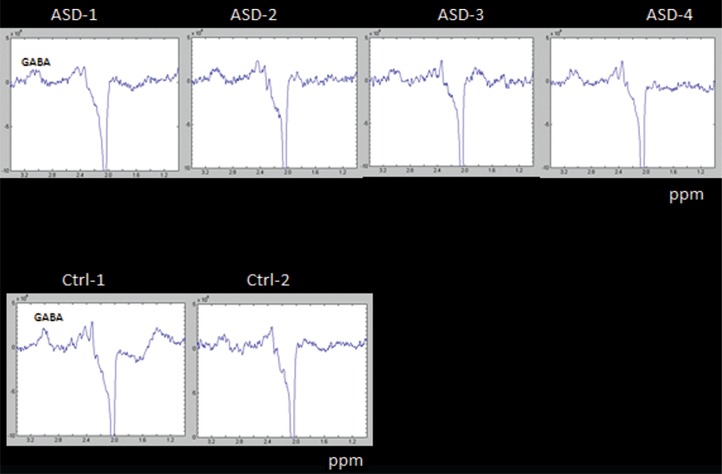
MRS measures of metabolites in four ASD children (Asd 1–4, upper panel) and two healthy control (Ctrl 1–2, lower panel) using a MEGA PRESS sequence, targeting GABA as an inhibitory neurotransmitter; ppm = parts per million.

The GABA results in Fig. 8 may be more promising with a clear difference in both response amplitude and envelope between the ASD children and one of the healthy control children (with a tendency in the same direction for the comparison with the other healthy control subject).

Ipser et al. (26) concluded that the data from their meta-analysis of MRS studies of ASD individuals provide ‘some evidence that ASD is characterized by age-dependent fluctuations in metabolite levels across the whole brain and at the level of specific regions thought to underlie ASD-associated behavioural and affective deficits’ (Abstract). A similar conclusion was reached by Kubas et al. (42) who investigated 12 children with autism, aged 8–15 years, and 16 control children without a autism diagnosis. The voxel for the MRS measurements were located in the frontal lobe, in the anterior cingulate region. The results showed reduced NAA/Cr, GABA/Cr, and Glu/Cr ratios in the autism group compared to the healthy control group.

## Summary and conclusions

The results showed differences in both neuronal activation and synaptic activity between ASD children and healthy controls. The differences were most profound in the frontal lobes and for GABA concentration. As such, the results could contribute to understanding the neurobiological underpinnings of ASD and ASD symptoms. The aim of the present article was to present the Bergen ASD project and to show some preliminary fMRI and MRS results from a limited sample that may point to new possibilities to link the brain and the gut, by providing data on neuronal and synaptic functioning in the normally functioning human brain. fMRI and MRS have given the scientist and clinician new windows into a complex but fascinating relationship of brain and gut functioning that could have important consequences for future treatment of ASD. Despite its promises, MR measures also provide serious challenges that have been highlighted in the article, thus cautioning against overinterpretation of findings that could be spurious relationships between variables. However, despite the limitations, we believe that fMRI and MRS will represent important tools for both diagnostics and treatment evaluations when it comes to ASD.
